# Forgotten Dreams: Recalling the Patient in British Psychotherapy, 1945–60

**DOI:** 10.1017/mdh.2015.4

**Published:** 2015-04

**Authors:** James Poskett

**Affiliations:** Department of History and Philosophy of Science, University of Cambridge, Free School Lane, Cambridge, CB2 3RH, UK

**Keywords:** Psychoanalysis, Dreams, Patients, Psychotherapy, Klein, Winnicott

## Abstract

The forgotten dream proved central to the early development of Sigmund Freud’s psychoanalytic technique in *The Interpretation of Dreams* (1900). However, little attention has been paid to the shifting uses of forgotten dreams within psychotherapeutic practice over the course of the twentieth century. This paper argues that post-war psychotherapists in London, both Jungian and Freudian, developed a range of subtly different approaches to dealing with their patients’ forgotten dreams. Theoretical commitments and institutional cultures shaped the work of practitioners including Donald Winnicott, Melanie Klein, Anna Freud, and Edward Griffith. By drawing on diaries and case notes, this paper also identifies the active role played by patients in negotiating the mechanics of therapy, and the appropriate response to a forgotten dream. This suggests a broader need for a detailed social history of post-Freudian psychotherapeutic technique, one that recognises the demands of both patients and practitioners.

## 1. Introduction

Patients undergoing psychotherapy routinely failed to remember their dreams. This much was consistent throughout the twentieth century. In *The Interpretation of Dreams*, published on the cusp of the 1900s, Sigmund Freud noted, ‘we are so familiar with the fact of dreams being liable to be forgotten, that we see no absurdity in the possibility of someone having had a dream in the night and of his not being aware in the morning’.^1^ Superficially similar remarks were made by the British psychoanalyst Donald Winnicott in the post-war period in which he stated, ‘some people never clearly remember their dreams’.^2^ Other psychotherapists of this era, such as the Jungian practitioner Edward Fyfe Griffith, made the same observation.^3^ Patients also directly recorded their difficulty in remembering dreams. In an unpublished dream diary from the late 1950s, a female patient undergoing psychotherapy in London penned the following: ‘I woke up in the night feeling afraid – yet I could not remember at all what I had been dreaming about.’^4^

This apparent continuity has led to a blind spot in the study of twentieth-century dream analysis. Ironically, Freud himself was well aware of the historical variation in approaches taken towards forgotten dreams. He summarises the diverse views of nineteenth-century writers such as Strümpell and Bonatelli, amongst others, in the opening chapter of *The Interpretation of Dreams*.^5^ More recently, Rhodri Hayward has shown how Victorian radicals and spiritualists read dreams as relatively transparent allegories, without the need for specialist interpretation to recover what may have been forgotten.^6^ Historians of the post-Freudian period, however, have been less discerning: there have been no histories of the twentieth century which suggest a divergence from Freud’s own position that a forgotten dream is ‘a product of resistance’.^7^ In fact, there is an unhelpful tendency in which the practices of the twentieth century are seen as a straightforward application of Freudian themes. Steven Kruger, sophisticated in his work on the Middle Ages, is typical, writing ‘we have largely followed Freud in his suggestion that the dream is the “royal road to … the unconscious”’.^8^

This paper seeks to redress the balance. I argue that psychotherapists in post-war London developed a range of subtly different approaches to dealing with forgotten dreams, reflecting diverse social and institutional pressures. Additionally, I demonstrate how, in response to forgotten dreams, patients played an active role in shaping the mechanics of therapy.

In his classic paper entitled ‘The Social History of Dreams’, Peter Burke describes how historians may study the content of a dream in terms of its social significance.^9^ However, Burke also hints at the importance of the changing practices of dreaming. For instance, he recounts how one early modern astrologer recommended sleeping with a Bible under the ear in the hope of conjuring spiritually significant dreams.^10^ Burke is also keen to remind us that we ‘do not have access to the dream itself’.^11^ Methodologically, this paper builds on Burke’s recommendation by treating dreams in terms of a social history of practice At the same time, I also take up Tyrus Miller’s suggestion to consider the ‘material form of dream reports’.^12^ As I show, even the simple act of underlining a word in a dream diary could provide a means for patients to assert themselves. This focus on social practice is in preference to an historiography in which dreams are treated as scientific objects.^13^ My methodology is both adopted and vindicated in light of my contention that patients played an active role in developing post-war dream analysis. Prior to the growth of sleep laboratories in the 1960s, both upper middle-class patients and their therapists relied on notions of dreaming diffuse in wider culture.^14^ Their roles did not depend on scientific concerns surrounding ‘objectivity’.

In this paper I consider the work of four psychotherapists, all based in London in the period between 1945 and 1960: Donald Winnicott, Melanie Klein, Edward Griffith and, to a lesser extent, Anna Freud. Winnicott, Klein and Anna Freud are well known so I will not repeat their biographies in detail here. Griffith, in contrast, is almost unknown in the history of psychotherapy. He is more often associated with histories of birth control and the marriage guidance movement, having co-founded the Marriage Guidance Council in 1943.^15^ He later fused these interests with psychological work, undergoing Jungian training in 1947 under Baroness Vera von der Heydt at the Davidson Clinic in Edinburgh.^16^ Throughout the 1950s he took on private adult patients for psychotherapeutic work at his Baker Street practice before publishing *Marriage and the Unconscious* in 1957, extolling the virtues of Jungian therapy for the resolution of marital problems.^17^ He attended meetings of the Society of Analytic Psychology, formed in 1945, and therefore represents a Jungian perspective in my historical analysis.

The time period and therapists identified in this study have been chosen for a number of reasons. First, the post-war era saw the concurrent emergence of both new forms of psychotherapy and new attitudes towards the patient. Following Sigmund Freud’s death in 1939, the British Psychoanalytic Society was beset with methodological conflict. Anna Freud and Melanie Klein’s ‘controversial discussions’ during the war years ultimately laid the ground for a three-fold division of the British Psychoanalytic Society: practitioners were separated into Freudians, Kleinians and the Middle Group.^18^ At the root of this division was a debate about how to practise psychotherapy. Broader changes within the medical profession were closely linked to these emerging techniques. London as a site is significant in this respect. In the wake of the Blitz, new institutions emerged charged with strengthening the relationship between the mother, the family and the state.^19^ Only in the post-war capital could one find a National Health Service (NHS) hospital, a Family Planning Association clinic, the headquarters of the Marriage Guidance Council and a psychoanalyst’s couch all a bus ride apart. In London, patients and practitioners met each other within a series of overlapping therapeutic cultures. Griffith, for instance, split his time between the Middlesex Hospital and his Baker Street clinic, just a twenty-minute walk away. In fact, a number of Griffith’s hospital patients originally presented with physiological complaints before taking up a course of private psychotherapy. One male patient in 1951 had been diagnosed with ‘a disease of the pancreas’ yet ‘said that his illness was a psychological one’. As Griffith explained, ‘the condition had been going on for some months and he was sent to various doctors in London’ before finally undergoing Jungian psychotherapy.^20^ Similarly, in 1950 Michael Balint set up a discussion group at the Tavistock Clinic with the hope of developing a psychoanalytically inspired approach to general practice.^21^ Winnicott often attended these sessions, meeting Balint in the evenings after seeing between five and six private patients during the day.^22^ Balint’s call for general practitioners within the new NHS to treat the ‘whole person’ is just one example of a broader trend in which British doctors came to recognise the importance of the ‘patient’s view’.^23^ The 1947 edition of Robert Hutchinson’s *Clinical Methods*, for instance, recommended doctors to ‘visualize the life of one’s patient sharing his emotions and viewing step by step his daily habit’.^24^ As John Forrester has argued, a close study of the post-war period can help to uncover the intimate relationship between ‘psychoanalysis and the changing character of the medical profession’ more generally.^25^ By studying the practices of the doctor–patient relationship in the context of forgotten dreams, this paper therefore contributes to a broader medical historiography in which the post-war period sees the emergence of a new kind of patient: one recognised as active and autonomous.^26^

Second, there is a wealth of archival material available to complement my study, mostly held at the Wellcome Library and British Psychoanalytic Society in London. These sources, a mixture of typed and hand-written case notes, allow me to reconstruct the practice of psychotherapy, rather than relying solely on published theoretical texts and cases. David Armstrong has, however, long warned against taking doctors’ descriptions of patients at face value.^27^ I therefore draw on substantial material produced by patients, particularly diaries. These sources, whilst treated carefully as the product of the therapeutic encounter, allow me to establish the active role played by patients with greater confidence.

Third, these psychotherapists represent a cross-section of theoretical backgrounds amongst a relatively small community operating within London during this period. Winnicott, Klein and Anna Freud represent emerging groups within the British Psychoanalytic Society, whereas Griffith provides a Jungian angle, an aspect often missing from histories of British psychotherapy.^28^ This range of theoretical positions is not intended to demonstrate the importance of psychological frameworks *per se*. Rather, it provides an opportunity to uncover the roles and limits of psychotherapeutic theory in practice. As I demonstrate, approaches to forgotten dreams relied on a composite of theoretical underpinning alongside individual therapists’ positions within certain social and institutional cultures. In fact, given the active role played by patients, responses to forgotten dreams had to serve both theoretical and pragmatic ends.

Pragmatically, dream reports and interpretations acted as a central medium in which patients and therapists communicated, operating as a space in which languages could develop in order to discuss topics deemed intensely private, such as sex and the body.^29^ Indeed, whether Freudian, Jungian or otherwise, psychotherapists considered sexual talk an essential ingredient of therapy. Griffith regularly complained of the ‘quite extraordinary ignorance that many couples have about ordinary physical matters’.^30^ However, recent oral histories have revealed that the typical middle-class patient would have lacked the requisite vocabulary of sexual fulfilment, at least in the sense of personal gratification and orgasm.^31^ Women in particular adopted a ‘practice of ignorance’ with respect to sexual knowledge: a performance designed to ensure propriety.^32^ Unsurprisingly, in cases lacking substantial dream material, both practitioners and patients found it hard to interact with one another.

The complete absence of dreams presented the most serious problem. Winnicott reports a male patient who ‘could not remember any dream’. This dearth of dream material then reinforced a developing lack of communication, Winnicott describing how the patient ‘said it would be a waste of time to go on talking’.^33^ The production of partially remembered dreams also had the potential to hinder therapy. In her case notes Klein describes a woman who ‘Had lots of dreams’ but could only remember the appearance of ‘3 3’s’ in one and ‘an arch’ in another. Klein then writes of the patient: ‘Great difficulty in associating. No association to the 3’s.’^34^ In the absence of this medium of communication, Klein’s attempts to continue with the patient prove problematic. She moves on to ask about the patient’s attraction to a certain married man but is cut short: ‘This topic seems most painful … says she cannot speak about it and breaks off there.’ Later in the analysis, once the poverty of dream material is overcome, the man comes to feature in the patient’s dreams and is then readily discussed: ‘She thinks he is very fond of her and she is of him. It might perhaps be quite nice to marry him.’^35^

As these cases illustrate, dream analysis did not diminish in importance during this period. However, the focus did change dramatically. The recovery of latent content, as in Freud’s original practice, became less of a priority. Instead, both psychotherapists and patients turned their attention to the pragmatic uses of dream analysis, rather than the content alone.^36^ Dreams could be used to discuss embarrassing problems, explore sexual feelings, or understand shifting marital roles. Forgotten dreams were therefore problematic in a new way. With limited dream reports, therapists and patients found it difficult to communicate with one another: developing strategies to proceed in their absence soon became critical to the practice of psychotherapy.

## 2. Melanie Klein

Klein takes what might be seen as the typical psychoanalytic route. In *Narrative of a Child Analysis* she attributes the absence of dreams to a form of psychological resistance on the part of her patient.^37^ The patient, ten-year-old Richard, reports that he cannot remember a dream. Klein concludes ‘[He] was trying to avoid feeling frightened by maintaining that it was only funny, and this was also why he had forgotten the dream and found it so difficult to report it’.^38^ She goes on to explain ‘Richard brought little material on that day. There were long breaks and he was obviously under the full impact of the depression preceding the parting’.^39^ Despite Klein’s appeal to ‘resistance’, her approach is not narrowly determined by psychoanalytic theories of repression. Rather, in response to Richard’s lack of dreams, Klein draws on a specific notion of depression which reflected her position both as a theorist and as a Viennese emigrant.

Across Europe throughout the nineteenth century, depression had been characterised by the medical profession as a general tendency towards gloomy thoughts and, specifically, it was thought to have an endogenous origin, arising from a weakened nervous system.^40^ Under the influence of continental psychiatrists such as Adolf Meyer, this concept shifted throughout the early twentieth century. Depression, at least in continental Europe, came to feature an exogenous component: reactions to the environment could induce states of psychological maladjustment. Meyer exemplified this view, developing the notion of ‘reaction types’ in the early 1900s.^41^ This binary view of depression, with both exogenous and endogenous modes, flourished in German-speaking countries. Freud’s own *Mourning and Melancholia* explicitly invoked ‘environmental influences’, comparing melancholia to mourning via ‘the reaction to the loss of a loved person’.^42^ Klein too developed her nascent theories of depression first in Berlin during the 1920s where she undertook her initial child analyses.^43^ These also drew on the notion of environmental influences. In Berlin, Klein identified two-year-old Rita’s depression as a reaction to the domestic environment, describing a shift in attitude when moving between the garden and nursery along with a ‘desire to be left alone in a room with her father’.^44^ These ideas developed into the 1940s in which Klein, still emphasising environmental influences, described the psychogenesis of depression as arising from ‘the slow process of testing reality … to renew the links to the external world’.^45^ In contrast, the binary division between endogenous and exogenous depression remained much more controversial in Britain. David Henderson and Robert Gillespie’s *Textbook of Psychiatry* exposed British students to Meyer’s reaction types but Aubrey Lewis, head of the Institute of Psychiatry from 1946 to 1966, still proved a particularly vocal critic.^46^ Klein’s approach, invoked in the absence of dream material, reflected her background in the broad German-speaking tradition of locating depression within a patient’s physical and emotional environment. This analysis of depression ultimately served two purposes for Klein. It provided a theoretical basis for approaching the absence of dream reports. It also had the pragmatic effect of provoking a reaction from the patient, providing further material to work with.

Klein’s theoretical attention to innate infant phantasies has led some historians to mistakenly discount her interest in the material conditions of social life.^47^ However, her deployment of depression in the absence of dreams reveals her close attention to the relationship between the domestic environment and family relations. In her analysis of Richard she identifies his depression as an emotional reaction to changing domestic circumstances. Following Richard’s failure to remember a dream, Klein begins to build a picture of Richard’s emotional world, locating it within his new home life. She describes his love of his old house in the city and a dislike of the countryside: ‘Richard was very sad because he loved their house, his room, the lounge, his train – all of it’. Klein then ties Richard’s feelings about his new domestic environment to his emotional attitude towards his parents, particularly the absence of his father: ‘Now that Richard again slept in the same room with Mummy, and Daddy was left alone, he felt that Daddy was thrown out and lonely’.^48^

This construction of an emotional perspective allowed Klein to account for the lack of dream material. In the case of Richard, the shifting family circumstances explained his difficulty in producing dream reports. But this approach also served Klein pragmatically. By presenting the patient with a construction of their own emotional viewpoint, she provided an opportunity for response. In doing so, Klein’s patients played an active role in negotiating the source of their emotional states, and so the causes of their lack of dreams. Richard, for instance, responded by picking out a drawing of an earlier dream: ‘He picked out Drawing 14 and said, with a telling look at Mrs K., “This is the worst of all.”’^49^ So, whilst Richard did not produce new dream material, he did provide Klein with an additional emotional reaction and so form of communication.

For other patients, Klein’s presentation of their own emotional viewpoint provoked a correction. An adult female patient, for example, recounts a partially remembered dream in which she reports appearing naked at school. Further details are scarce and Klein accounts for the lack of detail in terms of the patient’s emotional state: ‘this episode happened in front of other children … [she was] repelled by the thought of being undressed’. This view of the patient’s emotional world provokes a correction and, in this case, the production of further dream material: ‘She corrects my impression that this episode happened in front of other children – she was alone with the headmistress … why should she be so repelled by the thought of being undressed?’^50^ As in the case of Richard, Klein’s approach often had the effect of eliciting further material, restoring communication in the absence of a dream report.

## 3. Donald Winnicott

In contrast to Klein, Winnicott does not deploy the notion of depression. Instead he saw the lack of dream material as arising from the patient’s limited understanding of psychotherapeutic technique. In one example Winnicott introduces a patient by writing ‘He could not remember any dream belonging to this moment of sleep.’ Winnicott then describes his belief that patients are much more successful in recounting their dreams if they do so via an analyst, describing himself as ‘a suitable medium’.^51^ However, for Winnicott, the patient can only make use of the analyst if they understand his role. Winnicott then continues to attribute the successful production of dream material to the patient’s eventual comprehension of the mechanics of therapy:

I was able to go on to develop the theme of the analytic situation and together we worked out a rather clear statement of the specialized conditions provided by the analyst …. Following this the patient had a very important dream.^52^

This approach, focused on the relative knowledge of the patient, reflects Winnicott’s position at the intersection of specialist and popular psychotherapeutic culture. In early twentieth-century Britain, psychoanalysis was taken up as a topic of interest by middle-class magazines and newspapers. This fuelled a burgeoning market for non-specialist texts: Geraldine Coster’s *Psychoanalysis for Normal People*, first published in 1926, reached a third edition and was still available for purchase in the late 1940s.^53^ However, within the context of this popular reception, there was little differentiation between theories and practitioners: Jung’s universal unconscious and Freud’s dream analysis were simply blended together.^54^ Unlike Klein, Winnicott was heavily involved in this much broader cultural reception, appearing regularly on radio broadcasts following the Second World War. Indeed, Winnicott’s education at The Leys School and Jesus College, Cambridge furnished him with the requisite BBC radio broadcast intonation. (It is hard to imagine Klein’s thick Austrian accent going down so well.) Winnicott also regularly gave lectures to teachers, nurseries and mothers. In one such talk to a local mental health association, he reminded his audience that there are ‘many varieties of psychotherapy’ before attempting to enumerate some of the basic differences.^55^

**Figure 1:**
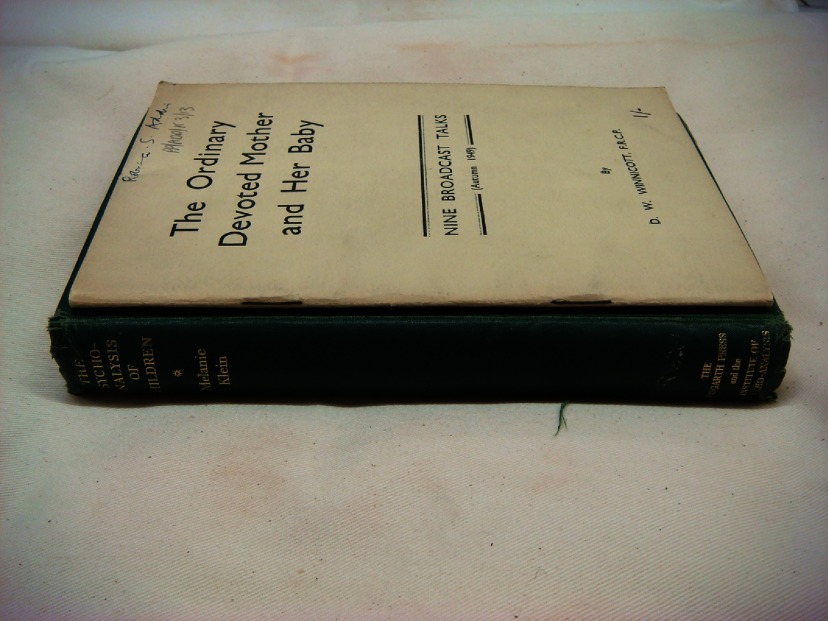
The material difference between Donald Winnicott’s pamphlet (top) and Melanie Klein’s Hogarth Press monograph (bottom), Wellcome Library, London.

In his approach to the lack of dreams, Winnicott draws on an appreciation that patients do not arrive with an understanding of the specifics of his therapeutic approach. He therefore sees the role of the analyst as something that must be established rather than assumed, particularly if dream material is to be forthcoming. A comparison of Winnicott and Klein’s published work illustrates this point. Klein does not expect her patients to read her dense, technically worded texts. They are produced almost exclusively for a professional psychoanalytic readership. In contrast, for Winnicott, publication could serve his aim to establish the role of the analyst as a ‘suitable medium’, an important prerequisite for the production of dream material. Winnicott’s *The Ordinary Devoted Mother and her Baby*, for instance, was published in conjunction with his radio broadcasts in the late 1940s (Figure 1). It was available freepost for a fraction of the cost of an average psychoanalytic text.^56^ This cheap pamphlet, widely distributed amongst his London patient base, then found its way into the analytic session. In such cases it provided a means by which Winnicott could induce a patient to reassess the role of the analyst. In a case note Winnicott reports ‘She had studied my Ordinary Devoted Mother papers again and had underlined the relevant passages and she knew that I really do understand what she needs’.^57^ In this instance, the material form of the pamphlet is put to use: the patient is able to return to the transient radio broadcast, underlining relevant passages.

In late 1947 another one of Winnicott’s female patients also reflected closely on the role of the analyst. Having difficulty discussing what she describes as a ‘trauma dream’, the patient writes, ‘this morning …. I felt I didn’t know about the next step, what to do with him as analyst or what relationship to make out of it’.^58^ But these first-hand accounts also reveal the active role patients played in negotiating the role of the analyst. Winnicott’s patients did not blindly accept the content of his publications. Rather, a number of patients saw a role for Winnicott in direct opposition to his theoretical view. Instead of acting as a medium for communication, patients requested that Winnicott remain silent in order that they may come to a personal understanding of their problems. The same female patient suffering from the ‘trauma dream’ went on to write, ‘I want to warn him that at any moment the thing I may need most from him is silence’.^59^ Such an approach once again reveals the influence of the early popularisation of psychoanalysis in Britain. Texts from this period explicitly contrasted the emphasis on self-discovery in psychoanalysis with the alleged imposition of interpretation found in hypnotism and mental suggestion.^60^ Patients’ appeals to ‘silence’ can therefore be understood in terms of a popular emphasis on self-discovery. Indeed, in his own case notes Winnicott acknowledged that ‘This feels to the patient like something that she has achieved, getting me to be silent’.^61^ Winnicott, of course, still believed his patients should see him as a ‘medium’ for communication, particularly in the absence of dream material. The relative worth of ‘silence’ was therefore contested by patient and therapist, with the pragmatic effect of eliciting an emotional response. Indeed, in one instance Winnicott breaks his silence to the effect of frustrating his patient:

On Monday I did say two things, and I said them not because I found it difficult to be silent but because I thought they ought to be said …. she took it that I had rebuked her.^62^

On another occasion, the mere sound of Winnicott folding his notes frustrates the patient into responding to him:

[The] patient reported to me that what I had done at the end of the hour on Friday was very disturbing …. It appears that as she got up to go there was a sound as if paper were being crinkled.^63^

In this instance, Winnicott goes on to analyse the basis of the frustration: ‘by making me not speak she is turning me into a woman, castrating me, making me impotent’.^64^ These accounts reveal the pragmatic effect of Winnicott’s approach to the lack of dream material. A focus on the role of the analyst provided patients with an opportunity to contest the mechanics of therapy. This produced emotional responses which then formed the basis of further work.

## 4. Edward Griffith

Griffith tackled the problem of limited dream material differently to both Klein and Winnicott. Crucially, he is the only therapist to actively encourage patients to write down and record their dreams. In a number of cases, patients even forwarded their dream material by post in advance of a session.^65^ Again, this served both theoretical and pragmatic ends.

From a theoretical perspective, Griffith understood dreams as a means by which patients could take ownership of their problems, writing of one male patient that ‘in a later dream … he is beginning to accept responsibility for his life, for what he has experienced and for what he has done’.^66^ For Griffith, writing down one’s dreams, or painting them, was an important step in achieving this goal, describing ‘the necessity for these people to learn to express their feelings through painting and dream’.^67^ Jung too, in contrast to Freud, had encouraged the recording of dreams as a means to take responsibility for one’s self:

I also show [patients] how to work out their dreams in the manner described, so that they can bring the dream and its context with them in writing to the consultation …. In this way the patient learns how to deal correctly with his unconscious without the doctor’s help.^68^

To an extent, then, Griffith’s approach does draw on his Jungian background. However, in tying together the practice of dream-recording and personal responsibility, Griffith both relied on and reinforced ideas developing within the marriage guidance movement in which he was situated.

In light of an increasing divorce rate, post-war political concerns had once again turned to the topic of marital breakdown. When the Royal Commission on Marriage and Divorce reported its findings in 1956, it placed renewed emphasis on ‘the duties and responsibilities of marriage’ which it contrasted to ‘personal satisfaction, reckless of the consequences to others’.^69^ As Nikolas Rose has noted, this emphasis on individual obligations permeated the Beveridge welfare state.^70^ Practical developments within the National Marriage Guidance Council, the sole provider of formal marriage counselling in the 1950s, reflected this mood. In 1955, the National Marriage Guidance Council hired a new training officer, John Wallis, who spearheaded a transition towards ‘non-directive’ counselling. This approach, whilst highly contested by the 1960s, reflected a view on behalf of the National Marriage Guidance Council that married couples needed to take personal responsibility for their problems.^71^ By the mid-1950s, the importance of personal responsibility was central to both political and therapeutic understandings of marriage.

**Figure 2:**
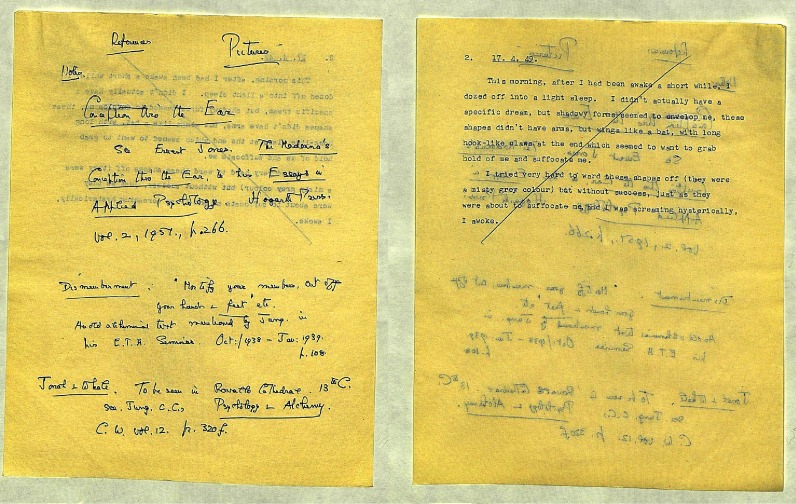
Edward Griffith prepares his lecture notes (left) on the back of a discarded dream (right). PP/EFG/B.126, Griffith Papers, Wellcome Library, London.

Griffith’s approach, influenced by both his Jungian background and contemporary approaches to marriage guidance, also had a pragmatic effect: the practice of recording dreams made physical what other therapists’ patients were prone to forget. By encouraging his patients to record their dreams, Griffith ensured that there would always be material to work with during any given session. In one case note Griffith reports a female patient who ‘hardly bothered to mention’ a dream. Despite the absence of dream material, Griffith continued with the therapy by reference to the patient’s previous writings.^72^ In fact, Griffith’s relative ease of access to material meant he often disregarded reports which consisted of partially remembered dreams. He even drafted his lecture notes on the back of dream reports he clearly thought uninteresting (Figure 2). These included references to forgotten or vaguely remembered dreams, one patient writing, ‘This morning, after I had been awake a short while, I dozed off into a light sleep. I didn’t actually have a specific dream’.^73^ In the presence of such an abundance of dream material, there was no particular pressure for Griffith to consider the significance of such testimony.

With this in mind, Griffith’s patients had an active role to play in establishing the relevance or otherwise of forgotten dreams. This was often achieved by making novel use of the writing practice encouraged by Griffith. One patient indicated which dreams she believed to be of particular significance by underlining passages in her dream diary. When she forwarded her dreams to Griffith, the patient also included a cover letter explaining the purpose of her practice: ‘have underlined the passages in these dreams which I think apply to these categories’.^74^ Other patients used this opportunity to analyse their own dreams prior to the analytic session. In the early 1950s, one of Griffith’s male patients sent a copy of his dream ‘together with his own analysis of it’. Again, this provided an opportunity to flag the importance of partially remembered dreams, the patient writing, ‘I feel that my girl and I have slowed up and got stuck. There seems to be some vague connection here with the dream. I couldn’t see where I was really going in the race and I think I’m facing the wrong way’.^75^

Similar writing tactics adopted by Griffith’s patients were characterised by a culture of dream-recording which had become fashionable outside psychotherapy earlier in the century. From the 1920s onwards, members of the British middle classes had taken to recording their dreams at the moment of waking. The popularity of this morning routine was reinforced following the publication of John Dunne’s hugely successful *An Experiment with Time* in 1927.^76^ This practice, with the notebook and pencil ‘kept under the pillow’, facilitated the recording of immediate physical and emotional reactions to the dream.^77^ As part of the same social group, Griffith’s patients appropriated this method for the purpose of psychotherapy, often recording additional information concerning the context of waking. This provided another means by which they could establish the relevance of certain dreams, even if only partially remembered. In 1949, for instance, one of Griffith’s patients recorded an extremely vague dream, writing only that ‘this dream was just like a short flash’. However, she then writes, ‘I was disturbed from my sleep... I awoke very annoyed’ and so asserts the importance of a dream Griffith might have otherwise discarded. The effect was such that this dream was collected together with others by Griffith for work on a potential publication.^78^

## 5. Anna Freud

Given the active role played by patients in responding to forgotten dreams, it is unsurprising to find that child analysis proved a particularly challenging domain. Indeed, early mental life was conceptualised in such a way that the question of young children forgetting dreams made little sense. Rather, they were thought not to recognise the difference between dreaming and waking life, Winnicott writing that ‘the waking life of an infant can be perhaps described as a gradually developing dissociation from the sleeping state’.^79^ Klein too subscribed to this view, reporting in her case notes that young children ‘cannot yet sufficiently understand what a dream or a thought of a day time or night time anxiety consist of’.^80^ Similarly, Anna Freud acknowledged that children often could not distinguish waking life from dream states. In an episode from the *Reports on the Hampstead Nurseries*, she recounts a child’s story of a bombing raid, concluding:

It is possible that all this talking represented the components of a dream which he had had at night. But it is also possible that this story had slowly prepared itself in him in the foregoing weeks.^81^

In this context, Anna Freud’s apparent lack of engagement with the problem of dream recollection, in contrast to solutions offered by Winnicott, Klein and Griffith, is best explained in light of her almost exclusive focus on child analysis following her time at the Hampstead Nurseries during the Second World War.

Nonetheless, forgotten dreams still had a role to play. With this understanding of childhood mental life in place, access to the ‘royal road’ was impeded. Therapists considered it problematic to proceed in the absence of clear-cut dream reports: Anna Freud, borrowing the language of delinquency, described ‘the average child’ as ‘wholly uncooperative’ with respect to the identification of dream material.^82^ Still, dream analysis did not decline in importance. Rather, in addressing this problem, therapists developed a range of new practices. As such, dreams (and the patients who forgot them) once again acted as a catalyst for further developments in psychotherapeutic practice. Therefore, whilst this paper has concentrated on direct responses to forgotten dreams, it also supports a much broader social history of post-Freudian technique: Anna Freud’s direct observation, Klein’s play therapy and Winnicott’s ‘squiggle’ game should all be read as the products of similar processes.^83^

## 6. Conclusion

In post-war London, diverse social, institutional and theoretical commitments produced a range of subtly different practices surrounding forgotten dreams. Crucially, these approaches were also actively shaped by patients, particularly in achieving the pragmatic task of restoring dream analysis as a medium of communication.

A number of broader conclusions concerning both the history of psychotherapy and the place of the patient in post-war medicine should be drawn from this study. First, as examples from Winnicott and Griffith demonstrate, the popularisation of psychoanalysis often fed back into practice. Well aware of this, some practitioners cultivated reading audiences in the hope of establishing a particular brand of therapy. Such strategies were only partially successful. Patients were rarely constrained by a single text, often reading across psychotherapists’ works, paying little attention to disciplinary boundaries. Even Klein’s patients, who were unlikely to have read her own books, drew on a range of authors: for instance, in the early 1950s one male patient argued that it was wrong to grant children too much freedom after having read Margaret Mead.^84^

Second, the advent of the post-war welfare state undoubtedly transformed the doctor–patient relationship in Britain. But this shift occurred in tandem with a range of new private services. In particular, the lack of substantial state support for long-term psychotherapy, contraception or marriage guidance ensured a considerable amount of cross-over between private and public practices.^85^ Nowhere was this more true than in London. Griffith, for instance, drew many of his own patients from the Middlesex Hospital at which he worked, whilst some of Klein’s patients were referred from military psychiatrists.^86^ This better explains how, precisely in this period, the contests over psychotherapeutic technique identified in this paper came to shape broader attitudes towards the patient within clinical medicine. However, whilst the ‘patient’s view’ certainly emerged in the common context of the welfare state and new psychoanalytic theories it was not a straightforward product of these two factors. As this paper demonstrates, patients too played an active role in securing a space in which they would be treated as autonomous individuals.

Third, the sheer diversity of approaches adopted towards forgotten dreams reinforces the need to study the development of post-Freudian technique in further detail. But, despite this diversity, it is noteworthy that all four practitioners, along with their patients, agreed on the significance of dreams and the problematic nature of forgetting. This, rather than a specific practice of dream analysis, appears to be Sigmund Freud’s enduring legacy in the years immediately after his death.^87^ Irrespective of their choice of therapist, patients began to agonise over their dreams. A final dreamer, one of Griffith’s female patients from the early 1950s, provides us with a closing image of this new emotional world:

Ever since I’ve had this dream I feel a bundle of nerves …. I’ve had to simply force myself to come here to-day, I’ve even cried at the thought of having to come.^88^

In post-war London, patients could no longer afford to ignore their dreams, and neither can we.

## Footnotes

1. SigmundFreud, The Interpretation of Dreams: The Standard Edition of the Complete Psychological Works of Sigmund Freud, Vol. 4 (London: Hogarth Press, 1953), 43.

2. DonaldWinnicott, ‘Primitive emotional development’, in Donald Winnicott (ed.), Collected Papers: Through Paediatrics to Psycho-Analysis (London: Tavistock, 1958), 145–156: 151.

3. EdwardGriffith, Marriage and the Unconscious (London: Secker & Warburg, 1957), 40.

4. PP/EFG/B.15: Paintings of a Woman Undergoing Analysis *c.1958, Griffith Papers, Wellcome Library, London (henceforth Griffith Papers)*.

5. Freud, *op. cit.* (note 1), 45, and Henri Ellenberger, *The Discovery of the Unconscious* (London: Penguin Press, 1970), 309.

6. RhodriHayward, ‘Policing Dreams: History and the Moral Uses of the Unconscious’, History Workshop Journal, 49 (2000), 142–160: 147.

7. Freud, *op. cit.* (note 1), 520.

8. StevenKruger, Dreaming in the Middle Ages (Cambridge: Cambridge University Press, 1992), 1.

9. PeterBurke, ‘L’histoire sociale des rêves’, Annales Économies, Sociétés, Civilisations, 28, 2 (1973), 329–342.

10. Burke, *op. cit.* (note 9), 334.

11. Burke, **op. cit.** (note 9), 333 (translation by the author).

12. TyrusMiller, ‘In the Blitz of Dreams: Mass-Observation and the Historical Uses of Dream Reports’, New Formations, 44 (2001), 34–51: 38–9.

13. Histories of both the pre- and post-Freudian period tend to focus on dreams as scientific objects, see Doris Kaufmann, ‘Dreams and selfconsciousness: mapping the mind in the late eighteenth and early nineteenth centuries’, in Lorraine Daston (ed.), *Biographies of Scientific Objects* (Chicago: University of Chicago Press, 2000), 67–85 and Kenton Kroker, *The Sleep of Others and the Transformations of Sleep Research* (Toronto: University of Toronto Press, 2007).

14. Kroker, *op. cit.* (note 13), 341.

15. JaneLewis, David Clark and David Morgan, ‘Whom God Hath Joined Together’: The Work of Marriage Guidance (London: Routledge, 1992), 60.

16. EdwardGriffith, The Pioneer Spirit (Upton Grey: Green Leaves Press, 1981), 102–105.

17. Griffith, *op. cit.* (note 3), 109.

18. PearlKing and Riccardo Steiner, *The Freud–Klein Controversies 1941–45* (London: Routledge, 1991).

19. NikolasRose, Governing the Soul (London: Routledge, 1991), 151.

20. EFG/B.13: Case of Hypoglycaemic Episodes with Possible Psychological Causes c.1951, Griffith Papers.

21. ThomasOsborne, ‘Mobilizing Psychoanalysis: Michael Balint and the General Practitioners’, Social Studies of Science, 23, 1 (1993), 175–200.

22. PP/DWW/K.7, Winnicott Appointment Diary 1955, Winnicott Papers, Wellcome Library, London (henceforth, Winnicott Papers).

23. MichaelBalint, The Doctor, His Patient, and the Illness (London: Pitman Medical Publishing Company, 1957), 7.

24. DavidArmstrong, ‘The Patient’s View’, Social Science and Medicine, 18, 9 (1984), 737–744: 738.672953310.1016/0277-9536(84)90099-6

25. JohnForrester, Dispatches from the Freud Wars: Psychoanalysis and its Passions (London: Harvard University Press, 1997), 203.

26. The idea of the patient as a ‘consumer’ emerges in the 1960s and 1970s, building on these earlier themes, Alex Mold, ‘Repositioning the Patient: Patient Organizations, Consumerism, and Autonomy in Britain during the 1960s and 1970s’, *Bulletin of the History of Medicine*, 87, (2013): 225-49.10.1353/bhm.2013.0022PMC375297523811711

27. Armstrong, *op. cit.* (note 24), 737–39.

28. ThomasKirsch, The Jungians: A Comparative and Historical Perspective (London: Routledge, 2000), 40–44.

29. The role of dreams as ‘communicative acts’ is noted in David Shulman and Guy Stroumsa, ‘Introduction’, in David Shulman and Guy Stroumsa (eds), *Dream Cultures: Explorations in the Comparative History of Dreaming* (Oxford: Oxford University Press, 1999), 3–16: 7.

30. Griffith, *op. cit.* (note 3).

31. KateFisher and Simon Szreter, Sex Before the Sexual Revolution (Cambridge: Cambridge University Press, 2010), 363.

32. KateFisher, Birth Control, Sex and Marriage in Britain, 1918–1960 (Oxford: Oxford University Press, 2008), 72.

33. DonaldWinnicott, ‘Withdrawal and regression’, in Donald Winnicott (ed.), Collected Papers, Through Paediatrics to Psycho-Analysis (London: Tavistock, 1958), 255–261: 256.

34. PP/KLE/B.74: Case Material: Patient Ju. 1948, Klein Papers, Wellcome Library, London (henceforth Klein Papers).

35. *Ibid*.

36. SusanBudd, ‘The shark behind the sofa: recent developments in the theory of dreams’, in Daniel Pick and Lyndal Roper (eds), Dreams and History: the Interpretation of Dreams from Ancient Greece to Modern Psychoanalysis (London: Routledge, 2004), 253–270: 261.

37. Klein’s *Narrative of a Child Analysis* is consulted despite being published in 1961. The case work took part during the early 1940s but the text itself was composed throughout the 1950s. It is therefore read as a product of the period considered.

38. MelanieKlein, Narrative of a Child Analysis (London: Karnac Books, 1996), 155.

39. *Ibid*., 448.

40. GermanBerrios, ‘Mood disorders’, in German Berrios and Roy Porter (eds), History of Clinical Psychiatry: Origin and History of Psychiatric Diseases (London: Athlone, 1993), 384–405: 386.

41. StanleyJackson, Melancholia and Depression (New Haven, CT: Yale University Press, 1986), 198.

42. SigmundFreud, Mourning and Melancholia: The Standard Edition of the Complete Psychological Works of Sigmund Freud, Vol. 14 (London: Hogarth Press, 1957), 243.

43. MelanieKlein, ‘The psycho-analytic play technique: its history and significance’, in Roger Money-Kyrle (ed.), The Writings of Melanie Klein, Vol. 3 (London: Karnac Books, 1993), 122–140: 124.

44. MelanieKlein, The Psychoanalysis of Children (London: Karnac Books, 1998), 3; Klein, *op. cit.* (note 43), 124.

45. MelanieKlein, ‘Mourning and its Relation to Manic-Depressive States’, The International Journal of Psychoanalysis, 21 (1940), 125–153: 136.

46. Jackson, *op. cit.* (note 41), 213.

47. Adam Phillips writes ‘Klein … makes little reference in her work to social or economic conditions’: Adam Phillips, *Winnicott* (London: Fontana, 1988), 44.

48. Klein, *op. cit.* (38), 214–5.

49. *Ibid*., 215.

50. PP/KLE/B.74: Case Material: Patient Ju 1948. Klein Papers.

51. Winnicott, *op. cit.* (note 2), 257.

52. *Ibid*., 257.

53. GrahamRichards, ‘Britain on the Couch: The Popularization of Psychoanalysis in Britain 1918–1940’, Science in Context, 13, 2 (2000), 183–230: 184–201.1162462810.1017/s0269889700003793

54. DeanRapp, ‘The Early Discovery of Freud by the British General Educated Public, 1912–1919’, Social History of Medicine, 3, 2 (1990), 217–243: 218.1162257910.1093/shm/3.2.217

55. DonaldWinnicott, ‘Varieties of psychotherapy’, in Clare Winnicott, Ray Shepherd and Madeleine Davis (eds), Deprivation and Delinquency (London: Tavistock, 1984), 232–240: 232.

56. *The Ordinary Devoted Mother and her Baby*cost 1s. 2d. in 1949. For comparison, a typical psychoanalytic title from the Hogarth Press, such as Anna Freud’s *The Psycho-Analytic Treatment of Children*, cost 10s. 6d. in 1947.

57. PP/DWW/F.2: Adult Clinical Material 1950s–1960s, Winnicott Papers.

58. P32: Anonymous Account of Treatment with D. W. Winnicott, British Psychoanalytic Society, London.

59. *Ibid*.

60. Richards, *op. cit.* (note 53), 193.

61. PP/DWW/F.2: Adult Clinical Material 1950s–1960s, Winnicott Papers.

62. *Ibid*.

63. *Ibid*.

64. *Ibid*.

65. PP/EFG/B88/2: Griffith: For Studies in the Symbolism of Dreams. Case material IV, Griffith Papers and PP/EFG/B20/2: Griffith Case Material: Dream Material – Misc. Patients, Griffith Papers.

66. PP/EFG/B17: Case Material: Account and Case of Man with Drawings, Griffith Papers.

67. PP/EFG/B126: Notes for Lecture on the Use of Art and Painting in Psychotherapy, Griffith Papers.

68. CarlJung, ‘The practical use of dream-analysis’, in Kathleen Raine (ed.), Dreams (London: Routledge, 2002), 87–108: 98.

69. Lewis, Clark and Morgan, *op. cit.* (note 15), 91–2.

70. NikolasRose, Governing the Soul (London: Routledge, 1991), 224.

71. Lewis, Clark and Morgan, *op. cit.* (note 15), 105.

72. PP/EFG/B.126: Notes for Lecture on Use of Art and Painting in Psychotherapy, Griffith Papers.

73. *Ibid*.

74. PP/EFG/B.88/2: Griffith: For Studies in the Symbolism of Dreams. Case material IV, Griffith Papers.

75. PP/EFG/B.20/2: Griffith case material ‘dream material’, Griffith Papers.

76. IanHacking, Historical Ontology (Cambridge, MA: Harvard University Press, 2002), 250.

77. JohnDunne, An Experiment with Time (London: Faber & Faber, 1934), 80.

78. PP/EFG/B.85/2: Griffith: For Studies in the Symbolism of Dreams. Case Material I, Griffith Papers.

79. Winnicott, *op. cit.* (note 2), 152.

80. PP/KLE/B.88: Klein: Patients’ Material. Dreams, Klein Papers.

81. AnnaFreud, Infants without Families and Reports on the Hampstead Nurseries, 1939–1945 (London: Hogarth Press, 1974), 64.

82. AnnaFreud, ‘The contributions of direct child observation to psychoanalysis’, in Anna Freud (ed.), Research at the Hampstead Child-Therapy Clinic and Other Papers 1956–1965 (New York: International Universities Press, 1969), 97–104: 101.

83. For the broader social history of psychoanalysis, see Eli Zaretsky, *Secrets of the Soul: A Social and Cultural History of Psychoanalysis* (New York: Knopf, 2004).

84. PP/KLE/B.75: Case material: Patient BA 1949–1950, Klein Papers.

85. No doctor in this period was obliged to give contraceptive advice under the National Health Service unless it was necessary for medical treatment, Audrey Leathard, *The Fight for Family Planning* (London: Macmillan, 1980), 80.

86. PP/KLE/B.75: Case material: Patient BA 1949-1950, Klein Papers; EFG B85/2: Griffith ‘For studies in the symbolism of dreams’ case material I, Griffith Papers; EFG/B13: Case of hypoglycaemic episodes with possible psychological causes *c.1951, Griffith Papers*.

87. As John Forrester argues for the early twentieth century, ‘there was no surer way of becoming a Freudian than by repeating Freud’s methods on one’s own dream’: John Forrester, ‘Remembering and Forgetting Freud in Early Twentieth-Century Dreams’, *Science in Context*, 19, 1 (2006), 65–85: 70.10.1017/s026988970500076117147216

88. EFG B85/2: Griffith ‘For studies in the symbolism of dreams’ case material I, Griffith Papers.

